# Acute and long-term grief reactions and experiences in parentally cancer-bereaved teenagers

**DOI:** 10.1186/s12904-021-00758-7

**Published:** 2021-05-27

**Authors:** Tove Bylund-Grenklo, Dröfn Birgisdóttir, Kim Beenaert, Tommy Nyberg, Viktor Skokic, Jimmie Kristensson, Gunnar Steineck, Carl Johan Fürst, Ulrika Kreicbergs

**Affiliations:** 1 Department of Caring Science, Faculty of Health and Occupational Studies, University of Gävle, SE-801 76 Gävle, Sweden; 2 Faculty of Medicine, Department of Clinical Sciences Lund, Oncology and Pathology, Institute for Palliative Care, Lund University and Region Skåne, Medicon Village, Hus 404B, 223 81 Lund, Sweden; 3 Ghent University & Vrije Universiteit Brussel (VUB), End-of-Life Care Research Group, Ghent, Belgium; 4 Department of Public Health and Primary Care, Ghent University, Ghent, Belgium; 5 MRC Biostatistics Unit, University of Cambridge, Cambridge, UK; 6 Department of Oncology-Pathology, Karolinska Institute, Division of Clinical Cancer Epidemiology, Stockholm, Sweden; 7 Faculty of Medicine, Department of Health Sciences, Lund University, Lund, Sweden; 8 Department of Oncology, Sahlgrenska Academy at the University of Gothenburg, Division of Clinical Cancer Epidemiology, Institute of Clinical Sciences, Gothenburg, Sweden; 9 Department of Caring Sciences, Ersta Sköndal Bräcke University College, Palliative Research Center, Stockholm, Sweden; 10 Department of Women’s and Children’s Health, Karolinska Institutet, Stockholm, Sweden

**Keywords:** Adolescents, Bereavement, Cancer, Grief, Loss, Mourning, Oncology, Parental death, Teenagers, Unresolved grief, Young adults

## Abstract

**Background:**

Previous research shows that many cancer-bereaved youths report unresolved grief several years after the death of a parent. Grief work hypothesis suggests that, in order to heal, the bereaved needs to process the pain of grief in some way. This study explored acute grief experiences and reactions in the first 6 months post-loss among cancer-bereaved teenagers. We further explored long-term grief resolution and potential predictors of having had “an okay way to grieve” in the first months post-loss.

**Methods:**

We used a population-based nationwide, study-specific survey to investigate acute and long-term grief experiences in 622 (73% response rate) bereaved young adults (age > 18) who, 6–9 years earlier, at ages 13–16 years, had lost a parent to cancer. Associations were assessed using bivariable and multivariable logistic regression.

**Results:**

Fifty-seven per cent of the participants reported that they did not have a way to grieve that felt okay during the first 6 months after the death of their parent. This was associated with increased risk for long-term unresolved grief (odds ratio (OR): 4.32, 95% confidence interval (CI): 2.99–6.28). An association with long-term unresolved grief was also found for those who reported to have been numbing and postponing (42%, OR: 1.73, 95% CI: 1.22–2.47), overwhelmed by grief (24%, OR: 2.02, 95% CI: 1.35–3.04) and discouraged from grieving (15%, OR: 2.68, 95% CI: 1.62–4.56) or to have concealed their grief to protect the other parent (24%, OR: 1.83, 95% CI: 1.23–2.73). Predictors of having had an okay way to grieve included being male, having had good family cohesion, and having talked about what was important with the dying parent.

**Conclusion:**

More than half of the cancer-bereaved teenagers did not find a way to grieve that felt okay during the first 6 months after the death of their parent and the acute grief experiences and reaction were associated with their grief resolution long-term, i.e. 6–9 years post-loss. Facilitating a last conversation with their dying parent, good family cohesion, and providing teenagers with knowledge about common grief experiences may help to prevent long-term unresolved grief.

**Supplementary Information:**

The online version contains supplementary material available at 10.1186/s12904-021-00758-7

*“I remember once waking up in the middle of the night with an excruciating pain in my heart. It was stabbing, aching and burning. If I had not been told that psychological pain can manifest itself in physical pain I would have thought that I was dying that night. The pain was so intense; I think my heart broke in thousands of pieces that night.*

*Today, eight years later, my heart is no longer in thousands of pieces – at least not for any longer period of time.”*

Quote from one of the participants, a daughter who at age 14 lost her mother [1](p.31).

## Introduction

Undoubtedly, for a child, one of the most devastating experiences is the early death of a parent, which can seriously affect their health and wellbeing [2–7] in the short and long term. Bereavement in children and adolescents has been shown to be associated with increased risk of suicide attempts [8, 9] and increased mortality [10, 11], and previous research based on the same study sample as the current study showed almost a doubled risk of self-injury in the first 6–9 years following teenage bereavement [12, 13]. While not all bereaved children and adolescents will face these negative outcomes of bereavement [14, 15], risk factors such as sudden, unexpected or traumatic [5] loss, parental depression [16] and poor family cohesion [17, 18] have been identified. Complicated or prolonged grief is another factor that has been shown to be associated with those negative health-related outcomes in bereaved children and adolescents [19, 20]. This includes symptoms such as separation distress, pre-occupation with thoughts about the deceased person, and difficulties in accepting the loss or in returning to normal functioning after the loss [21–23]. It should be noted that the categorization of complicated or prolonged grief is still debated, particularly in children and adolescents [24, 25].

In bereaved adults, the characteristics of the grieving process are considered to be of importance for their wellbeing after bereavement [26–28]. In the immediate phase after bereavement, grief may often include powerful emotions such as shock, numbness, crying, anxiety and anger [29, 30] and many theories concerning coping with or recovering from loss, regardless of whether they focus on stages [11] or tasks [12], include the notion that, in order for the person to heal, they must deal with the pain in so-called “grief work”. Since Freud first came forward with this notion, the understanding of what “working through the grief” entails has changed over time [31], challenging the assumption that “grief work” is only a cognitive process of confronting the loss [31]. This can be seen in one of today’s relevant grief-theories, the Dual Process Model, stating that it is part of the normal grieving process for people to shift in and out of the intense emotional reaction to loss, described as oscillation between loss- and restoration-oriented grief reactions [32]. The Dual Process model was initially designed to understand conjugal bereavement [31] and yet more research is needed to build up the empirical evidence among bereaved children and teenagers. Grief is a unique experience and is highly influenced by individual traits, the relationship with the deceased and the circumstances surrounding the death, as well as social and cultural factors [25], and grief reactions among children and teenagers can differ from adults’ reactions [33]. Children and teenagers can often only tolerate the emotional pain for a short period of time compared with adults, shifting between intense feelings such as yearning, sadness or anger to rapidly returning to normal activities [33–35]. It has been highlighted that more knowledge is needed about the grieving process of children and teenagers [36–38] and many experts in the field seem to agree that not all knowledge from the adult bereavement research field can be transferred directly to children and teenagers [24]. Knowledge on various grief reactions of children and teenagers can be helpful for both bereaved children and their parents while dealing with the loss [39–41]. Nevertheless, for the last decades, the focus within the bereavement literature has mostly been on what is sometimes referred to as “pathological grief responses” among children and teenagers, while more research is also needed to better understand “normative grieving processes “ [25]. While knowledge regarding e.g. the needs of, and the meaning of grief in bereaved children, adolescents and young adults are increasingly being documented [42–44], we still need more knowledge about the youths’reactions and experiences of grief, in the immediate phase and long-term [24, 37].

To be able to provide more knowledge and to reduce suffering among parentally bereaved teenagers, more research based on teenagers’ own experience is needed to describe their normative and pathological grieving processes [25] both during the acute bereavement phase and long-term.

In the preparatory interviews with cancer-bereaved youths that were performed for this research project, the parentally bereaved informants described a range of different grief reactions in the immediate post-loss phase. Some concluded that they had not found “an okay way to grieve” (data not published).

The aim of this exploratory population-based study was to investigate 1) the prevalence of a set of grief experiences and reactions in the acute bereavement phase, i.e. the first 6 months post-loss, and 2) their possible associations with unresolved grief long-term, 6–9 years after the loss of a parent to cancer, as self-assessed by cancer-bereaved youths. Further, we explored the associations between demographic, family, and health care-related factors, and the experience of having had an okay way to grieve in the first 6 months post-loss.

## Method

### Study design and study population

We conducted a population-based nationwide survey in 2009–2010 in young adults who, during their teenage years, had lost a parent to cancer. The Swedish Cause of Death Register identified the individuals who had died from cancer at an age younger than 65 (based on International Classification of Diseases, 10th revision (ICD-10), codes C00–C96) in 2000–2003. This information was then used by the Multi-Generation Register to identify children who were bereaved of a parent between the ages of 13 and 16 and who had been living with both parents at the time of the loss. Because of the great variation in maturity levels during the teenage years [45], we decided to restrict this study to the youngest group of teenagers that would match grades 7 to 9 in the Swedish middle school.

For inclusion, the participants had to be living in Sweden at the time of the survey, be fluent in Swedish, and have an identifiable telephone number; also, their other parent still had to be alive. Altogether, 851 bereaved former teenagers were confirmed eligible for the study. All participants were between 18 and 26 years old at the time of the data collection. More details on the study protocol have been published elsewhere [46].

### Data collection

At the beginning of the data collection, each participant first received an introductory letter explaining the study objective. A questionnaire was sent only to those who, during a subsequent informative telephone call, consented to participate. Participants were informed both orally and in writing about their right to withdraw from the study at any time. The questionnaires were returned in pre-stamped envelopes, separately from the response cards in order to ensure anonymity. After a few weeks, a combined thank you and reminder card was posted, followed by reminder telephone calls to those whose responses were missing.

### Questionnaire development

A study-specific questionnaire was developed based on semi-structured interviews with 15 cancer-bereaved youths, and interviews with three health care professionals specialized in grief and palliative care, as well as the bereavement literature. To ensure that the questions we constructed were understood as intended, we tested the face validity of the questionnaire and response options with 15 cancer-bereaved former teenagers (six previously interviewed and nine newly invited individuals) in think-aloud interviews. Questionnaire development followed well-established routines that have been previously described [47, 48]. The final questionnaire contained 271 question items, set in different time frames, i.e. childhood, teenage years (before and after the loss), and young adulthood (at the time of the survey). A total of 37 items were considered relevant for this study.

### Measurements

We used six single-item questions [49] to assess coping styles, grief experiences, expressions and behaviours in the acute bereavement phase, i.e. during the first 6 months after the loss of a parent (hereafter referred to as “*Acute grief experiences and reactions”*). These questions all started with “For the first half-year after your loss, would you agree with the statement: …” , followed by:

- “I had a way to grieve that felt okay.” (hereafter labelled: *Had an okay way to grieve,* or as its negative counterpart; *Did* *not* *have an okay way to grieve (R)* for its reversed form)
- “I clenched my teeth, built a wall around me and lived on as if nothing had happened.” (*Numbing and postponing*)
- “I withheld my grief to protect my other parent.” (*Concealed grief*)
- “The grief was so strong it felt as if I would not survive, as if I was going crazy or was not normal.” (*Overwhelmed by grief*)
- “People stopped me from grieving by drawing away when I was sad or praising me when I was being strong.” (*Discouraged from grieving*)
- “There was pressure from others that I should be more sad than I was showing.” (*Pressured to grieve*)

The response options for all abovementioned questions were: “Completely agree”, “Moderately agree”, “Slightly agree” and “Do not agree at all”.

Long-term grief resolution, i.e. at the time of the survey (6–9 years after the loss), was measured with the single-item question:

- “Have you worked through your grief?”, with the response options “No, not at all”, “Yes, a little”, “Yes, moderately” and “Yes, completely”.

This single-item question was well understood by bereaved participants in the face-validity interviews and has been used in previous studies [17, 50–53]. In a study on young adults, cancer-bereaved of a sibling, this question was validated against three questions from the Inventory of Complicated Grief (ICG), and found to be strongly correlated to them [53].

Additionally, we used ten demographic variables (e.g. gender of the child), three family-related variables (e.g. family cohesion), and 15 health care-related variables (e.g. teenagers’ level of trust in the health care provided to the dying parent in the final week of life) in our data analysis.

### Data analysis

The responses to all of the items measuring the grief experiences and reactions during the acute bereavement phase (the first 6 months post-loss) were dichotomized into “Agree” (moderately, and completely agree) and “Disagree” (slightly agree, and do not agree at all). The responses “No” and “Yes, a little” to the question of having worked through grief were labelled as “Unresolved grief” while “Yes, moderately” and “Yes, completely” were labelled as *having worked through grief*.

The relationship between the six acute grief experiences and reactions in the first 6 months following the loss, and perceived *unresolved grief* at follow-up was evaluated in terms of odds ratios (ORs). The unadjusted estimates were calculated using logistic regression which was then subsequently adjusted for three groups of possible confounders. The adjustment scheme applied decomposes into two steps. In the first step, all of the available possible confounders were classified as belonging to one of the classes “background variables”, “family-related variables” and “health care-related variables”. Within each group a logistic regression with a forward selection procedure was performed, using the variables as predictors of “unresolved grief at follow-up”. Selection was based on likelihood ratio *p*-values, with a *p*-value of 0.25 used as a stopping criterion. This means that the selection procedure was aborted if none of the remaining candidate variables were associated with a *p*-value of 0.25 or less when included in the model. Prior to each selection all individuals with missing values on any of the variables within a particular group of variables were excluded. In the second step, the groups of variables selected by the forward selection procedures were sequentially used to calculate the adjusted ORs with 95% confidence intervals (CIs).

A further analysis of the data was performed, where both crude and adjusted ORs were calculated again with the same three groups of possible confounders as before, but now with the data stratified by the gender of the participants.

In order to assess what variables might be associated with *Having had an okay way to grieve* in the 6 months following the loss, all variables considered in the previous analysis were treated as potential predictors of this outcome in bivariable logistic regression models. Once again likelihood ratio *p*-values were used to evaluate their predictive performance. The significant variables (*p*-value < 0.05) were subsequently used in conjunction as predictors in a multivariable logistic regression model, in order to investigate the effect of correlations among them on their significance as predictors.

## Results

A total of 851 cancer-bereaved youths (teenagers at the time of their loss) were confirmed eligible, 622 (73%) of whom returned the questionnaire. Fifty-four per cent of participants had lost their father and 46% had lost their mother. The characteristics of the participants are presented in Table 1.

**Table 1:** Characteristics of the study population

	N (%)
**Confirmed eligible**^**1**^	**851 (100)**
Unreachable	55 (6)
Declined to participate	66 (8)
Agreed initially but did not return the questionnaire	108 (13)
**Provided information**	**622 (73)**
**Gender of the participants**	
Male	309 (50)
Female	312 (50)
Not stated	1
**Year of birth (age, in years, at the time of the survey)**	
1988–1990 (19–21)	210 (34)
1986–1987 (22, 23)	286 (46)
1984–1985 (24–26)	123 (20)
Not stated	3
**Birth order**	
Oldest child	144 (23)
Middle child	148 (24)
Youngest child	302 (49)
Only child	27 (4)
Not stated	1
**Living arrangement and marital status**	
Lives with parent, is single	134 (22)
Lives with parent, has a partner (living apart)	70 (11)
Has moved away from parent, is single	153 (25)
Has moved away from parent, has a partner (living apart)	86 (14)
Lives with partner or spouse	176 (28)
Not stated	3
**Highest level of education attained (at the time of the survey)**	
Not applicable, never graduated	6 (1)
Middle school (≤9th grade)	49 (8)
High school (≥10th grade)	501 (81)
College/university	54 (9)
Other type of studies	11 (2)
Not stated	1
**Current employment status**^**2**^	
Studying at high school level	24/614 (4)
Adult education at high school level	31/613 (5)
Studying at university level	187/613 (30)
Employed or self-employed	355/616 (58)
Unemployed	91/616 (15)
On parental leave	9/613 (2)
On sick leave	7/613 (1)
**Residential area**	
Rural	54 (9)
Small village or town	113 (18)
Medium-sized town	283 (46)
City of more than 500,000	166 (27)
Not stated	6
**Gender of the deceased parent**	
Male	337 (54)
Female	284 (46)
Not stated	1

^1^ Confirmed eligible = all those identified in registers who met the inclusion criteria

^2^ More than one response alternative could be selected for this question. Number of responses per answer is provided

### Prevalence of the different acute grief experiences and reactions

Among the participants, 57% reported that they *had* *not* *had an okay way to grieve (R)*, as can be seen in Table 2. The most often agreed with out of the remaining five acute grief reactions were *numbing and postponing* (42%), *concealed grief* to protect the other parent (25%) and *being overwhelmed by grief* (24%). A total of 79 participants, or 13%, disagreed with all of the statements regarding grief during the acute bereavement phase.

**Table 2:** Prevalence of acute grief experiences and reactions (in the first 6 months post-loss) (*N* = 622)

For the first half-year after your loss, would you agree with the statement (*see phrasing in italics below*):	Do not agree N (%)	Slightly agree N (%)	Moderately agree N (%)	Completely agree N (%)	Missing N^**1**^
**DID NOT HAVE AN OKAY WAY TO GRIEVE**^**2**^ *“I did* *not* *have a way to grieve that felt okay.”*	107/614 (17)	158/614 (26)	227/614 (37)	122/614 (20)	8
**NUMBING AND POSTPONING** *“I clenched my teeth, built a wall around me and lived on as if nothing had happened.”*	117/616 (19)	239/616 (39)	148/616 (24)	113/616 (18)	6
**CONCEALED GRIEF** *“I witheld my grief to protect my other parent.”*	266/615 (43)	199/615 (32)	97/615 (16)	53/615 (9)	7
**OVERWHELMED BY GRIEF** *“The grief was so strong it felt as if I would not survive, as if I was going crazy or was not normal.”*	280/616 (45)	186/616 (30)	89/616 (14)	61/616 (10)	6
**DISCOURAGED FROM GRIEVING** *“People stopped me from grieving by drawing away when I was sad or praising me when I was being strong.”*	349/613 (57)	171/613 (28)	60/613 (10)	33/613 (5)	9
**PRESSURED TO GRIEVE** *“There was pressure from others that I should be more sad than I was showing.”*	328/616 (53)	172/616 (28)	78/616 (13)	38/616 (6)	6

^1^ Individuals with missing data are excluded from the prevalence calculations

^2^ To facilitate comparisons and avoid double negations, we here present the variable “I had a way to grieve that felt okay” as its negative counterpart,

“I did not have a way to grieve that felt okay”

### Associations between the acute grief reactions and long-term unresolved grief

Forty-five per cent of the participants reported not having worked through their grief at the time of the survey 6–9 years post-loss. Table 3 shows the associations between the six acute grief experiences and reactions in the first 6 months post-loss and reported unresolved grief 6–9 years later (i.e. at the time of the survey). The participants reporting not *having had an okay way to grieve (R)* were statistically significantly more likely to report *long-term unresolved grief* (OR: 4.32, 95% CI: 2.99–6.28). Statistically significant associations with unresolved grief long-term were also found in those who reported to have been *numbing and postponing* (OR: 1.73, 95% CI: 1.22–2.47), to have been *overwhelmed by grief* (OR: 2.02, 95% CI: 1.35–3.04), to have been *discouraged from grieving* (OR: 2.68, 95% CI: 1.62–4.56) and to have *concealed their grief* to protect the surviving parent (OR: 1.83, 95% CI: 1.23–2.73). All these associations remained statistically significant after controlling for the selected possible confounding demographic variables, family-related variables and health care-related variables (Table 3).

**Table 3:** Acute grief experiences and reactions, and the association with long-term unresolved grief

	RATIOS	ODDS RATIOS (ORs)	ODDS RATIOS ADJUSTED FOR BACKGROUND VARIABLES^**1**^	ODDS RATIOS ADJUSTED FOR BACKGROUND AND FAMILY-RELATED VARIABLES^**2**^	ODDS RATIOS ADJUSTED FOR BACKGROUND, FAMILY AND HEALTH CARE-RELATED VARIABLES^**3**^
	**N unresolved grief**^**4**^**/N grieving style (%)**	**OR (95% CI)**	**OR (95% CI)**	**OR (95% CI)**	**OR (95% CI)**
**DID NOT HAVE AN OKAY WAY TO GRIEVE**					
Entire group Agree	187/290 (64)	**4.32 (2.99–6.28)**	**4.23 (2.91–6.22)**	**4.19 (2.88–6.16)**	**4.14 (2.77–6.23)**
Entire group Disagree	69/233 (30)	1.0 (ref)	1.0 (ref)	1.0 (ref)	1.0 (ref)
Male participants Agree	75/118 (64)	**5.85 (3.41–10.25)**	**6.23 (3.59–11.07)**	**6.64 (3.79–11.98)**	**6.72 (3.65–12.84)**
Male participants Disagree	31/135 (23)	1.0 (ref)	1.0 (ref)	1.0 (ref)	1.0 (ref)
Female participants Agree	112/172 (65)	**2.95 (1.77–4.96)**	**2.94 (1.75–4.98)**	**2.84 (1.68–4.85)**	**2.73 (1.54–4.89)**
Female participants Disagree	38/98 (39)	1.0 (ref)	1.0 (ref)	1.0 (ref)	1.0 (ref)
**NUMBING AND POSTPONING**					
Entire group Agree	120/210 (57)	**1.73 (1.22–2.47)**	**1.68 (1.17–2.40)**	**1.66 (1.16–2.38)**	**1.57 (1.07–2.30)**
Entire group Disagree	137/315 (43)	1.0 (ref)	1.0 (ref)	1.0 (ref)	1.0 (ref)
Male participants Agree	48/94 (51)	**1.8 (1.08–3.03)**	**1.83 (1.09–3.09)**	**1.91 (1.13–3.25)**	**2.08 (1.16–3.79)**
Male participants Disagree	59/161 (37)	1.0 (ref)	1.0 (ref)	1.0 (ref)	1.0 (ref)
Female participants Agree	72/116 (62)	1.59 (0.98**–**2.61)	1.51 (0.92–2.50)	1.58 (0.95–2.63)	1.43 (0.82–2.48)
Female participants Disagree	78/154 (51)	1.0 (ref)	1.0 (ref)	1.0 (ref)	1.0 (ref)
**CONCEALED GRIEF (TO PROTECT MY LIVING PARENT)**					
Entire group Agree	80/133 (60)	**1.83 (1.23–2.73)**	**1.71 (1.14–2.60)**	**1.64 (1.08–2.53)**	**1.56 (1.00–2.45)**
Entire group Disagree	177/391 (45)	1.0 (ref)	1.0 (ref)	1.0 (ref)	1.0 (ref)
Male participants Agree	25/43 (58)	**2.19 (1.13–4.31)**	**2.27 (1.16–4.50)**	**2.55 (1.27–5.19)**	**3.41 (1.55–7.77)**
Male participants Disagree	82/211 (39)	1.0 (ref)	1.0 (ref)	1.0 (ref)	1.0 (ref)
Female participants Agree	55/90 (61)	1.41 (0.84–2.37)	1.45 (0.86–2.47)	1.20 (0.69–2.09)	1.04 (0.57–1.88)
Female participants Disagree	95/180 (53)	1.0 (ref)	1.0 (ref)	1.0 (ref)	1.0 (ref)
**OVERWHELMED BY GRIEF**					
Entire group Agree	81/131 (62)	**2.02 (1.35–3.04)**	**1.85 (1.21–2.86)**	**1.81 (1.18–2.80)**	**1.88 (1.19–2.98)**
Entire group Disagree	175/393 (45)	1.0 (ref)	1.0 (ref)	1.0 (ref)	1.0 (ref)
Male participants Agree	17/28 (61)	**2.35 (1.06–5.40)**	**2.54 (1.13–5.94)**	**2.73 (1.20–6.45)**	**3.22 (1.29–8.34)**
Male participants Disagree	90/227 (40)	1.0 (ref)	1.0 (ref)	1.0 (ref)	1.0 (ref)
Female participants Agree	64/103 (62)	1.56 (0.95–2.59)	1.59 (0.96–2.67)	1.53 (0.91–2.59)	1.64 (0.95–2.88)
Female participants Disagree	85/166 (51)	1.0 (ref)	1.0 (ref)	1.0 (ref)	1.0 (ref)
**DISCOURAGED FROM GRIEVING**					
Entire group Agree	54/78 (69)	**2.68 (1.62–4.56)**	**2.37 (1.41–4.08)**	**2.31 (1.37–3.99)**	**2.45 (1.42–4.32)**
Entire group Disagree	203/445 (46)	1.0 (ref)	1.0 (ref)	1.0 (ref)	1.0 (ref)
Male participants Agree	9/17 (53)	1.60 (0.59–4.39)	1.67 (0.61–4.64)	1.75 (0.64–4.92)	2.1472 (0.72–6.54)
Male participants Disagree	98/237 (41)	1.0 (ref)	1.0 (ref)	1.0 (ref)	1.0 (ref)
Female participants Agree	45/61 (74)	**2.76 (1.49–5.32)**	**2.79 (1.50–5.41)**	**2.65 (1.41–5.19)**	**2.74 (1.41–5.53)**
Female participants Disagree	105/208 (50)	1.0 (ref)	1.0 (ref)	1.0 (ref)	1.0 (ref)
**PRESSURED TO GRIEVE**					
Entire group Agree	46/91 (51)	1.08 (0.69–1.69)	1.07 (0.68–1.71)	1.04 (0.65–1.66)	1.10 (0.67–1.80)
Entire group Disagree	211/433 (49)	1.0 (ref)	1.0 (ref)	1.0 (ref)	1.0 (ref)
Male participants Agree	18/39 (46)	1.22 (0.61**–**2.43)	1.25 (0.62–2.51)	1.30 (0.64–2.62)	1.76 (0.79–3.92)
Male participants Disagree	89/216 (41)	1.0 (ref)	1.0 (ref)	1.0 (ref)	1.0 (ref)
Female participants Agree	28/52 (54)	0.91 (0.50–1.68)	0.93 (0.50–1.73)	0.87 (0.47–1.64)	0.85 (0.44–1.68)
Female participants Disagree	122/217 (56)	1.0 (ref)	1.0 (ref)	1.0 (ref)	1.0 (ref)

Acute grief experiences and reactions: first 6 months post-loss. Long-term unresolved grief: 6–9 years post-loss. Agree: moderately agree and completely agree; Disagree: do not agree and slightly agree

Variables retained after the logistic regression in the forward selection procedure, using the variables as predictors of unresolved grief, with selection being based on likelihood ratio *p*-values and the entry criterion of *P* < 0.25: ^*1*^*Odds ratio adjusted for background variables***:** gender (in the entire group, not used in the gender-stratified data analysis), age at loss. ^2^*Odds ratio adjusted for family-related variables***:** worried about the surviving parent. ^3^*Odds ratio adjusted for health care-related variables***:** the teenager’s perception of the health care professionals’ efforts to cure the parent; the teenager’s perception of the health care professionals’ efforts to prolong the parent’s life; whether the family had been given end-of-life information about the disease, treatment and death by a physician; whether the teenager had talked with their dying parent about what was important; awareness time at which the teenager realized that the parent would die from the disease; awareness time at which the teenager realized that death was imminent (hours or days)

^4^Missing values for unresolved grief (not included in the analyses): *n* = 63; demographic variables: *n* = 89; family-related variables: *n* = 13; health care-related variables: *n* = 115. Missing values are due to participants’ response of “I don’t know or remember” to selected variables. *CI* Confidence interval. *OR* Odds ratio

### Gender-stratified analysis

**Fig. 1:**
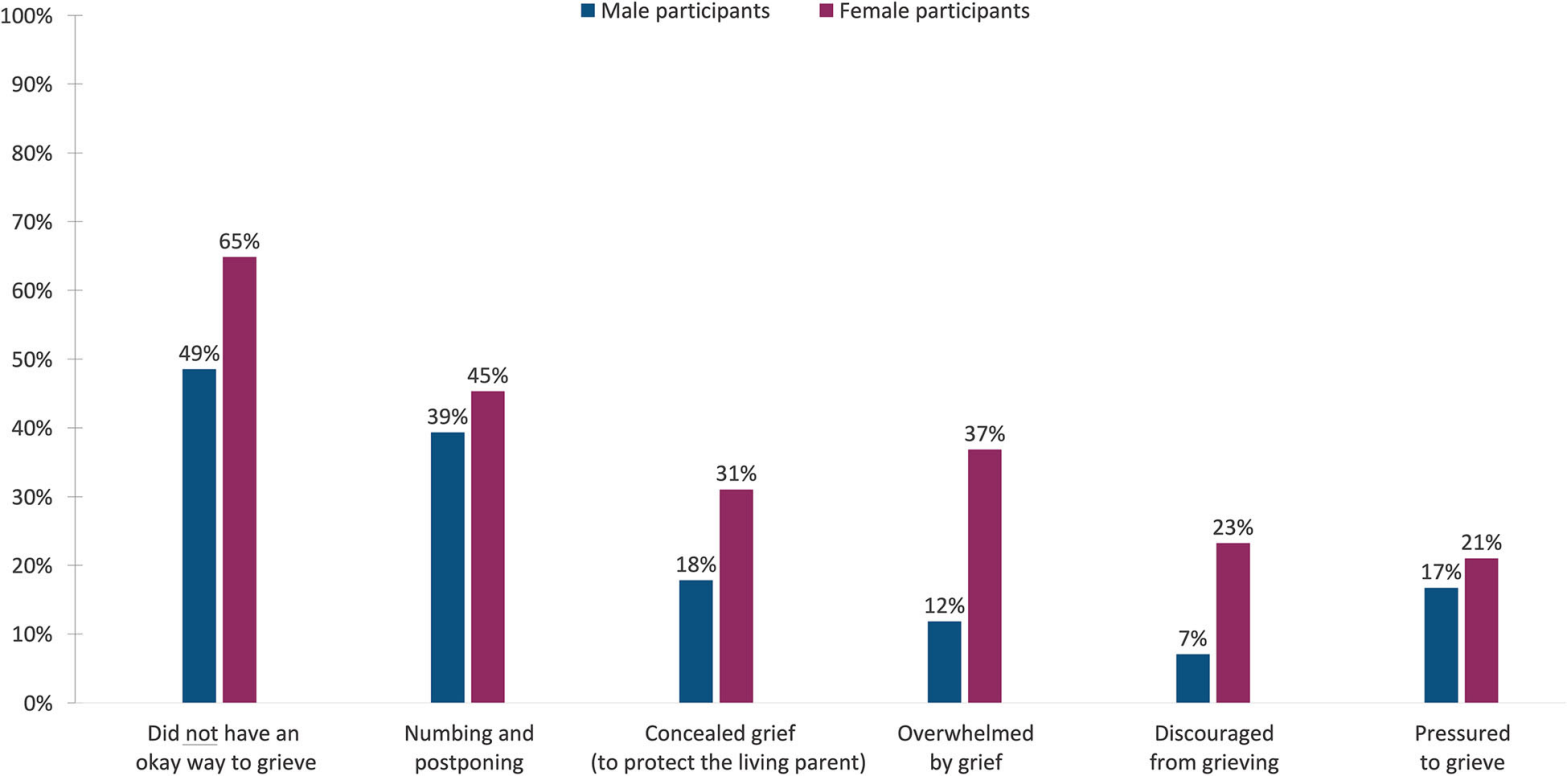
Prevalence of the six acute grief experiences and reactions in the first 6 months post-loss

**Fig. 2:**
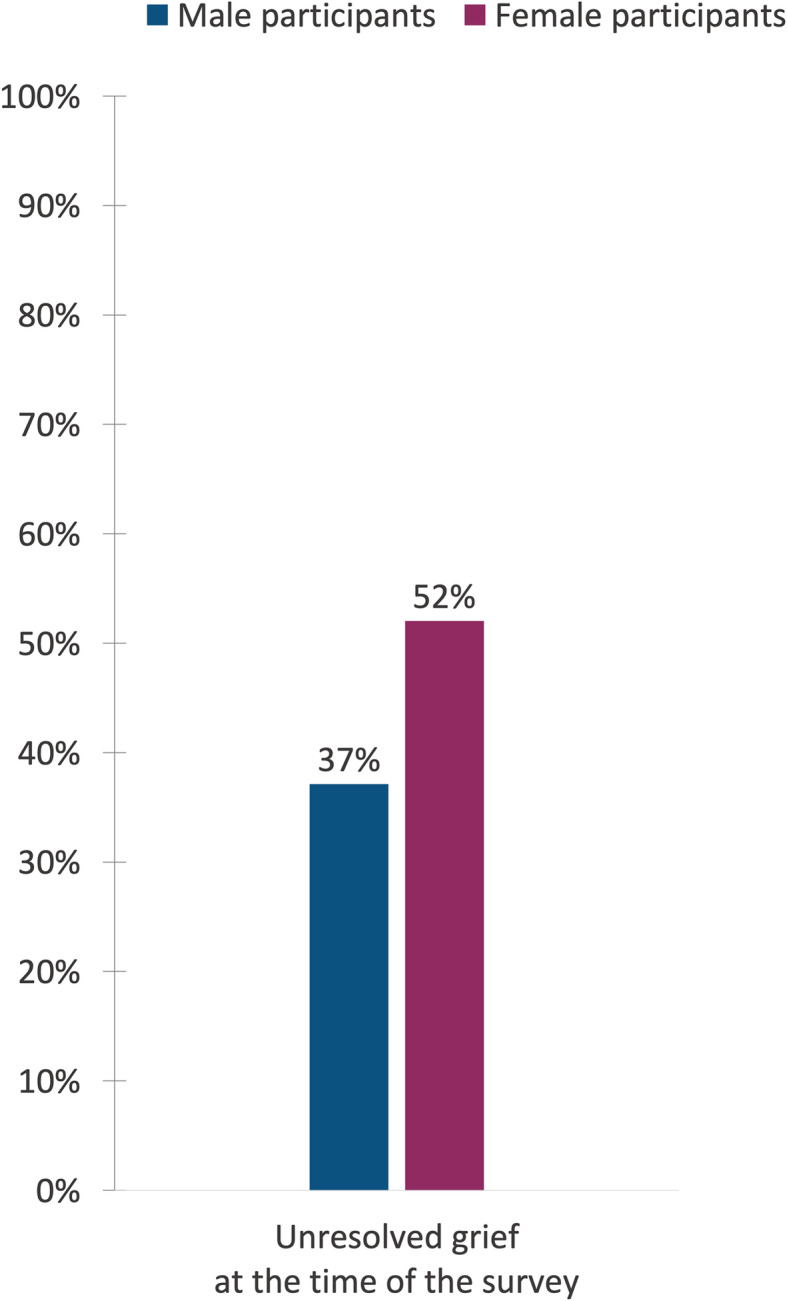
Prevalence of long-term grief resolution at the time of the survey (6–9 years post-loss)

Forty-nine per cent of the parentally cancer-bereaved male participants and 65% of the female participants reported *not* *having had a way to grieve that felt okay (R)* to them during the acute bereavement phase. Figure 1 illustrates the reported prevalence of the different grief experiences and reactions during the acute bereavement phase, subdivided by gender. Figure 2 shows the reported prevalence of grief resolution 6–9 years after the loss of a parent, where 37% of the male and 52% of the female participants reported long-term unresolved grief.

In further analysis of the data stratified by gender, *not* *having had an okay way to grieve (R)* was found to be statistically significantly associated with long-term unresolved grief in cancer-bereaved youths, both male (OR: 5.9, 95% CI: 3.4–10.3) and female (OR: 2.9, 95% CI: 1.8–5.0). These associations remained significant for both genders throughout the adjustments. However, only among the male participants were *numbing and postponing* (OR: 1.73, 95% CI: 1.22–2.47), *overwhelmed by grief* (OR: 2.02, 95% CI: 1.35–3.04) and *concealed grief* (OR: 2.19, 95% CI: 1.13–4.31) statistically significantly associated with long-term unresolved grief. These associations either remained or were strengthened after adjustments (Table 3). For female participants, the association with *long-term unresolved grief* was found for those reporting having been *discouraged from grieving* (OR: 2.76, 95% CI: 1.49–5.32). The association remained statistically significant and more or less unchanged after adjustments for background, and family and health care-related variables.

### Possible predictive factors for having had an okay way to grieve in the first 6 months post-loss

Nine out of 28 background, family, and health care-related variables were found to be statistically significantly associated with *having had an okay way to grieve*, based on the results of a univariate logistic regression (see Supplementary Table). These nine variables were then used in a multivariable logistic regression model (Table 4), where three of them were found to be statistically significantly associated with *having had an okay way to grieve*. *Male participants* were more likely to *have had an okay way to grieve* (OR: 1.77, 95% CI: 1.23–2.54). Those who stated that there was a *good family cohesion* during the first 6 months after the loss (OR: 2.17, 95% CI: 1.27–3.84), and those who reported that they *had talked with the dying parent about what was important* were more likely to *have had an okay way to grieve* than those who did not and wished they had (OR: 2.00, 95% CI: 1.35–2.97).

**Table 4:** Associations between possible predictive variables and having had an okay way to grieve in the first 6 months post-loss

	N who had had an okay way to grieve/N of individuals in the category (%)	OR (95% CI) of having had an okay way to grieve^**1**^	***P***-value^**1**^
**Gender of participants**			**0.0020**
Male	156/303 (51)	**1.77 (1.23–2.54)**	
Female	109/310 (35)	1.0 (ref)	
**Family cohesion during the teenage years, until the loss**			0.1459
Good (moderate, or very much cohesion)	254/563 (45)	1.86 (0.81–4.68)	
Poor (no, or a little cohesion)	9/48 (19)	1.0 (ref)	
**Family cohesion during the first 6 months after the loss**			**0.0046**
Good (moderate, or very much cohesion)	239/502 (48)	**2.17 (1.27–3.84)**	
Poor (no, or a little cohesion)	23/109 (21)	1.0 (ref)	
**Worried about the surviving parent the first 6 months after the loss**			0.0817
No (no, or a little worry)	104/206 (50)	1.40 (0.96–2.05)	
Yes (moderate, or very much worry)	161/407 (40)	1.0 (ref)	
**The teenager’s level of trust in the care provided to the dying parent in the final week of life**			0.5463
Trust (moderate, or very much trust)	218/485 (45)	1.19 (0.68–2.08)	
Distrust (no, or a little trust)	34/103 (33)	1.0 (ref)	
**The teenager’s perception of the health care professionals’ efforts to cure their parent**			0.3045
Good efforts (moderate, or very much)	212/451 (47)	1.37 (0.75–2.54)	
Poor efforts (no, or a little)	52/160 (32)	1.0 (ref)	
**The teenager’s perception of the health care professionals’ efforts to prolong the parent’s life**			0.6002
Good efforts (moderate, or very much)	211/459 (46)	0.84 (0.43–1.62)	
Poor efforts (no, or a little)	53/152 (35)	1.0 (ref)	
**The teenager’s perception of the health care professionals’ efforts to prevent the parent’s suffering**			0.1760
Good efforts (moderate, or very much)	240/524 (46)	1.54 (0.82–2.94)	
Poor efforts (no, or a little)	24/86 (28)	1.0 (ref)	
**The teenager had talked with their dying parent about what was important**			**0.0015**
Yes	118/225 (52)	**2.00 (1.35–2.97)**	
No, but I didn’t feel a need to	52/100 (52)	**1.79 (1.08–2.97)**	
No, and I wish I had	92/280 (33)	1.0 (ref)	

^1^Multivariable model of background, family and health care-related variables that were statistically significantly associated

(*p* < 0.05) in the bivariable analysis with having had an okay way to grieve

Missing values: 53 individuals were excluded because of missing values for any of the variables included in the model

*CI* Confidence interval; *OR* Odds ratio

## Discussion

This exploratory nationwide population-based study of 622 parentally bereaved former teenagers shows that more than half of the participants *had* *not* *found an okay way to grieve* in the first 6 months after the loss. *Not* *having had an okay way to grieve*, and four out of the five other acute grief experiences and reactions, including *numbing and postponing*, *concealing the grief* to protect the surviving parent, and being *overwhelmed by grief*, were associated with long-term unresolved grief. Differences were found between male and female participants in their reported grief experiences and reactions during the acute bereavement phase. Male participants, those who had talked with the dying parent about what they perceived as important, and those who had good family cohesion after the loss were more likely to *have had an okay way to grieve* in the immediate post-loss phase.

To the best of our knowledge, this is the first documentation of the prevalence or even existence of a number of different acute grief experiences and reactions post-loss in bereaved teenagers and the association of these experiences with long-term grief resolution. While some of them (e.g. *numbing and postponing, concealed grief,* or being *overwhelmed by grief*) are known reactions to loss and have been mentioned, in some form, in other studies [26, 27, 29, 30, 54–56], this is, as far as we know, the first study where teenagers were asked if they had found a way to grieve that felt okay to them. These thoughts were expressed by the bereaved teenagers themselves in the preparatory interviews and therefore included in the study-specific questionnaire.

Why so many of the parentally bereaved teenagers in our study seem to have been struggling with finding a way to grieve that felt okay to them during the acute bereavement phase is unclear to us. One possible explanation could be linked to the quality of their relationship with the surviving parent, where warmth and connection as well as positive parenting skills have been shown to benefit the children [2, 16, 25, 57–60]. Also, how the surviving parent is coping with their own grief has been shown to have an impact on their children’s grief reactions and ability to cope with the loss [59, 61–64]. Cancer, as the cause of death, has been found to significantly impact the risk of complicated grief among the bereaved [65], and may also be an explanation. Another possible explanation to consider, might be related to lack of experience and knowledge about common grief reactions in young people. More knowledge about what to expect after the death of a parent has been requested by bereaved teenagers and their surviving parents [39–41] and identified as helpful in their grieving process. It is also possible that more support is required according to individual needs after the death of a parent [66].

*Numbing and postponing* the grief was prevalent among the participants of our study. Although coping strategies that involve avoiding or suppressing emotions have been linked to psychological problems in bereaved children and teenagers and an open expression of grief is encouraged [67], it has also been argued that numbing and postponing grief can be an important part of their way to handle the grief [67]. Teenagers’ developmental stage can make them especially vulnerable to emotional stressors [68] and they are often only capable of dealing with the emotional pain for a short period of time [33–35]. The Dual Process Model describes an oscillation which is viewed as a normal part of the grieving process, allowing the person to move in and out of intense grief, and thus enabling them to deal with the loss in small doses at a time [31, 32]. We do not know whether the reported *numbing and postponing* among our participants was part of oscillating coping as described by the Dual Process Model, enabling them to handle their emotional pain from the grief, or whether they were putting their grief reactions on hold for a longer period of time.

Out of the six different acute grief reactions and experiences in our study, *not* *having had an okay way to grieve* was the factor that had the strongest association with long-term unresolved grief*.* A study of bereaved adults [29] found that having negative interpretations of one’s own grief reactions had a strong association with bereavement distress and symptoms of traumatic grief, even when those grief reactions are generally considered to be part of a normal grieving process [29]. This highlights the importance of encouraging or supporting bereaved teenagers to find a way of coming to terms with their own grief reactions.

Further analysis, based on the gender of the participants, showed that the female participants had a higher prevalence of all the different acute grief experiences and reactions compared to the male participants, and the female participants were more likely to report unresolved grief 6–9 years after the loss. In addition, we also found different acute grief reactions to be associated with unresolved grief in the cancer-bereaved male and female participants. However, *not* *having had an okay way to grieve* was found to be statistically significantly associated with unresolved grief in both genders.

There could be many reasons behind the identified gender differences. Although the literature on teenagers’ grief reactions is still limited, previous research has shown differences in grief reactions between the genders, where girls have been shown to report more persistent grief responses than boys and to be more likely to have prolonged grief disorder than boys [69]. It has also been reported that both normative and problematic grief responses decline more slowly in girls than in boys [69]. Regardless of why these differences in experience between the genders occur, we could assume, based on our findings, that there might be a need to approach teenage boys and girls differently during the acute bereavement phase.

Apart from the association between being male and *having had an okay way to grieve* during the acute bereavement phase, we also found an association between good family cohesion after the loss and *having had an okay way to grieve* during the acute bereavement phase. This is in line with previous research where family function, including family cohesion, was shown to impact children’s way of coping with loss [2, 4] and where bereavement support, with focus on improving the family function after the loss of a parent, was shown to be beneficial for children and teenagers [69–71].

We also found that those who had talked with the dying parent about what they perceived as important were more likely to *have had an okay way to grieve* in the acute post-loss phase. The vast majority of cancer-bereaved teenagers want to be told about the ill parent’s impending death [72] and being prepared for the loss of a parent has been shown to be of importance for children’s adjustments after the loss [41]. In families where children are able to openly communicate about their parent’s death, the children tend to adapt better in bereavement [73]. Children and teenagers have highlighted the importance of having the opportunity to say goodbye [39, 66, 74, 75] and those who were unable to have their final talk with their dying parent have reported resentment and sadness during their grief [76, 77]. However, for them to be able to have this opportunity, it is important for the health care personnel to communicate to the family, including the teenage offspring, when the death of a parent is near.

### Strengths and limitations

This population-based survey was conducted with a large sample, using study-specific questions based on preparatory interviews with, and tested for face validity in, the target group. It also included measurement of a number of potential confounders. This, together with a high response rate (73%) and the data collection method (with self-reported data collected directly from the former teenagers themselves, thus providing direct insight into the grief experiences and reactions of our target group) are the major strengths of our study.

Among the limitations, which should be considered when interpreting our results, are that we have no knowledge about the possible impact that unknown confounders or the responses from non-participants could have had on the results. That is, we do not know if those who declined participation in the study had more or less difficulties with grief than those who participated (i.e. potential selection bias). Not using standardized grief-measurements can be seen as a limitation. However, our intention was to study the participants’ subjective grief experiences and we believe using global single-item questions, directly asking about the real-life phenomena under investigation, can also be considered a strength. The questions were well understood by all of the bereaved participants in the face-validity interviews in this and other studies [50–52] and in this case it allowed us to collect a comprehensive data on teenagers’ own subjective experience when losing a parent to cancer.

Because of our study design, i.e. cross-sectional, we cannot rule out the possibility of recall-induced bias and that current grief resolution may have partly influenced some participants’ self-assessment of past events and circumstances. However, for ethical and practical reasons, collecting this data prospectively in a cohort study design was not possible. Instead, we had to mimic a longitudinal study design by anchoring the questions in childhood, teenage years, pre and post loss and today (at the time of the survey). In addition, we cannot know for what length of time our participants experienced the reported grief reactions and experiences, i.e. whether their answers reflected the whole first 6 months post-loss or whether the reactions occurred for a shorter part of that time. It is also noteworthy that in our exploratory study we found that 13% of the participants disagreed with all six of the statements regarding grief experiences, indicating a need to further explore other possible grief experiences and reactions that were not captured here.

Also, the eligibility criteria limit the generalizability of our findings to other groups such as teenagers from single-parent households, newly arrived immigrants or children outside the age range of this study.

### Implications

To be able to adjust to life after loss, both teenagers and their parents may benefit from knowledge of what to expect and the variety of grief reactions [39, 40]. The findings from our study indicate that it is important not to impose specific expectations on how the teenager’s grief should or should not be expressed or dealt with. Rather, we should try to gain a deeper understanding of how the young person experiences their own reactions and if they are okay with that or not. The findings that more than half of the participants did *not* *have a way to grieve that felt okay to them* during the acute bereavement phase and that many of them felt the need to suppress or conceal their grief to protect others, highlight the importance of attending to the needs of bereaved teenagers and encouraging them to find a way to grieve that feels okay to them. Further research probing deeper into what constitutes an okay way to grieve and what does not would be useful.

Regarding clinical implications, pre-loss communication between health care professionals and the family might facilitate the possibility to say goodbye. Health care professionals should be aware of the impact of good family cohesion and communication [37, 40, 41, 78] and facilitate it when a parent is seriously ill or dying, as this may potentially prevent long-term unresolved grief in bereaved youth. This could e.g. mean providing information about various grief reactions, screening for families and teenagers at risk for complicated grief and provide support as needed, such as through the Family bereavement program [69] or its Swedish adaptation; The Grief and Communication Family Support Intervention [79]. Apart from the role health care professionals can have in bereavement support it is also important to take more of a public health approach [80, 81]. Public awareness about the impact of social support, not only from the family but also from e.g. school professionals and peers [82, 83] may improve the wellbeing of bereaved youth.

## Conclusion

More than half of the parentally bereaved participants had not found a way to grieve that felt okay to them during the acute bereavement phase. This, as well as several of the acute grief experiences and reactions measured, was associated with unresolved long-term grief. Having had an okay way to grieve in the immediate post-loss phase was predicted by male gender, good family cohesion and having had a last conversation with the dying parent. Pre- and post-loss communication between health care professionals and the family, including the teenage children, about the imminent death, and about common acute grief experiences and reactions, normalizing the sometimes abysmal emotions that may be experienced, could facilitate coping with grief in the acute phase of bereavement, thus possibly reducing the risk of unresolved long-term grief.

## Supplementary Information

**Additional file 1: Supplementary Table.** Associations between background, and family and health care-related variables and having had an okay way to grieve in the first 6 months post-loss.

## Data Availability

The datasets generated and/or analysed during the current study are not publicly available due to legal and ethical restrictions as described by the Swedish law and ethical boards regarding data of sensitive nature, but are available from the corresponding authors on reasonable request. This is in order to assure data confidentiality and to protect the privacy of the research participants.
