# Correction to: Acute and long-term grief reactions and experiences in parentally cancer-bereaved teenagers

**DOI:** 10.1186/s12904-021-00797-0

**Published:** 2021-06-28

**Authors:** Tove Bylund-Grenklo, Dröfn Birgisdóttir, Kim Beernaert, Tommy Nyberg, Viktor Skokic, Jimmie Kristensson, Gunnar Steineck, Carl Johan Fürst, Ulrika Kreicbergs

**Affiliations:** 1 Department of Caring Science, Faculty of Health and Occupational Studies, University of Gävle, 801 76 Gävle, Sweden; 2 Faculty of Medicine, Department of Clinical Sciences Lund, Oncology and Pathology, Institute for Palliative Care, Lund University and Region Skåne, Medicon Village, Hus 404B, 223 81 Lund, Sweden; 3 End-of-Life Care Research Group, Ghent University & Vrije Universiteit Brussel (VUB), Ghent, Belgium; 4 Department of Public Health and Primary Care, Ghent University, Ghent, Belgium; 5 MRC Biostatistics Unit, University of Cambridge, Cambridge, UK; 6 Department of Oncology-Pathology, Division of Clinical Cancer Epidemiology, Karolinska Institute, Stockholm, Sweden; 7 Faculty of Medicine, Department of Health Sciences, Lund University, Lund, Sweden; 8 Department of Oncology, Division of Clinical Cancer Epidemiology, Sahlgrenska Academy At the University of Gothenburg, Institute of Clinical Sciences, Gothenburg, Sweden; 9 Department of Caring Sciences, Palliative Research Center, Ersta Sköndal Bräcke University College, Stockholm, Sweden; 10 Department of Women’s and Children’s Health, Karolinska Institutet, Stockholm, Sweden

**Correction to: BMC Palliat Care 20, 75 (2021)**

**https://doi.org/10.1186/s12904-021-00758-7**

Following publication of the original article [1], the authors identified an error in the author name of Kim Beernaert.

The incorrect author name is: Kim Beenaert.

The correct author name is: Kim Beernaert.

The author group has been updated above and the original article [1] has been corrected.
