# Supplementary material for: Acute and long-term grief reactions and experiences in parentally cancer-bereaved teenagers
Source: BMC Palliat Care. 2021 May 27;20:75. doi: 10.1186/s12904-021-00758-7 (PMC8161967; doi:10.1186/s12904-021-00758-7)
Supplement: Supplementary file 1 — Additional file 1: Supplementary Table. Associations between background, and family and health care-related variables and having had an okay way to grieve in the first 6 months post-loss. [file 12904_2021_758_MOESM1_ESM.docx]

**Supplementary Table. Associations between background, and family and health care-related variables and having had an okay way to grieve in the first 6 months post-loss**

|  | **N of having had an okay way to grieve/N of individuals in the category (%)** | **OR (95% CI) of having had an okay way to grieve^1^** | **P-value^1^** |
| --- | --- | --- | --- |
| **BACKGROUND VARIABLES** |  |  |  |
| **Gender of participants** |  |  | **<0.0001** |
| Male | 156/303 (51) | 1.96 (1.42–2.71) |  |
| Female | 109/310 (35) | 1.0 (ref) |  |
| **Birth year of the participant** |  |  | 0.8966 |
| 1984–1985 | 53/120 (44) | 1.02 (0.65–1.60) |  |
| 1986–1987 | 119/283 (42) | 0.93 (0.65–1.34) |  |
| 1988–1990 | 91/208 (44) | 1.0 (ref) |  |
| **Year of loss** |  |  | 0.5566 |
| 2000 | 73/163 (45) | 0.97 (0.63–1.50) |  |
| 2001 | 63/141 (45) | 0.97 (0.62–1.52) |  |
| 2002 | 51/134 (38) | 0.74(0.46–1.17) |  |
| 2003 | 75/165 (45) | 1.0 (ref) |  |
| **Age at loss** |  |  | 0.3005 |
| 13 | 50/120 (42) | 1.0 (ref) |  |
| 14 | 66/157 (42) | 1.02 (0.63–1.65) |  |
| 15 | 82/166 (49) | 1.37 (0.85–2.20) |  |
| 16 | 62/157 (39) | 0.91 (0.56–1.48) |  |
| **Birth order** |  |  | 0.8996 |
| Oldest child | 64/143 (45) | 1.0 (ref) |  |
| Middle child | 64/143 (45) | 1 (0.63–1.59) |  |
| Youngest child | 125/300 (42) | 0.88 (0.59–1.32) |  |
| Only child | 12/27 (44) | 0.99 (0.42–2.26) |  |
| **Gender of the dead parent** |  |  | 0.2582 |
| Male | 150/331 (45) | 1.20 (0.87–1.66) |  |
| Female | 115/282 (41) | 1.0 (ref) |  |
| **Birth year of the dead parent** |  |  | 0.6021 |
| 1960–1969 | 33/79 (42) | 1.0 (ref) |  |
| 1955–1959 | 69/173 (40) | 0.92 (0.54–1.59) |  |
| 1950–1954 | 74/158 (47) | 1.2 (0.71–2.13) |  |
| 1949 or before | 78/174 (45) | 1.13 (0.66–1.95) |  |
| **Birth year of the living parent** |  |  | 0.4758 |
| 1960–1969 | 42/104 (40) | 1.0 (ref) |  |
| 1955–1959 | 81/188 (43) | 1.12 (0.69–1.82) |  |
| 1950–1954 | 81/195 (42) | 1.05 (0.65–1.71) |  |
| 1949 or before | 52/104 (50) | 1.48 (0.85–2.56) |  |
| **Education level of the living parent** |  |  | 0.8998 |
| Middle school | 47/113 (42) | 1.0 (ref) |  |
| High school | 108/247 (44) | 1.09 (0.70–1.72) |  |
| College/university | 94/224 (42) | 1.02 (0.64–1.61) |  |
| **Education level of the dead parent** |  |  | 0.3735 |
| Middle school | 53/112 (47) | 1.0 (ref) |  |
| High school | 06/235 (45) | 0.91 (0.58–1.44) |  |
| College/university | 94/234 (40) | 0.75 (0.47–1.18) |  |
| **FAMILY-RELATED VARIABLES** |  |  |  |
| **Family cohesion during the teenage years, until the loss** |  |  | **0.0002** |
| Good (moderate, or very much cohesion) | 254/563 (45) | 3.56 (1.77–7.97) |  |
| Poor (no, or a little cohesion) | 9/48 (19) | 1.0 (ref) |  |
| **Family cohesion during the 6 months after the loss** |  |  | **<0.0001** |
| Good (moderate, or very much cohesion) | 239/502 (48) | 3.40 (2.11–5.67) |  |
| Poor (no, or a little cohesion) | 23/109 (21) | 1.0 (ref) |  |
| **Worried about the surviving parent,**  **first 6 months after the loss** |  |  | **0.01** |
| No (no, or a little worry) | 104/206 (50) | 1.56 (1.11–2.19) |  |
| Yes (moderate, or very much worry) | 161/407 (40) | 1.0 (ref) |  |
| **HEALTH CARE-RELATED VARIABLES** |  |  |  |
| **Cancer type** |  |  | 0.9923 |
| Central nervous system | 26/66 (39) | 1.0 (ref) |  |
| Breast, gynaecological | 49/116 (42) | 1.13 (0.61–2.10) |  |
| Gastro-intestinal | 57/127 (45) | 1.25 (0.69–2.31) |  |
| Urinary tract, prostate | 13/29 (45) | 1.25 (0.51–3.03) |  |
| Skin, sarcoma | 17/43 (40) | 1.01 (0.45–2.20) |  |
| Haematological | 18/39 (46) | 1.32 (0.59–2.94) |  |
| Lung, head and neck, thyroid | 36/85 (42) | 1.13 (0.59–2.19) |  |
| Unknown primary | 4/12 (33) | 0.77 (0.19–2.71) |  |
| Don’t know, don’t remember | 26/58 (45) | 1.25 (0.61–2.57) |  |
| **Disease recurrence** |  |  | 0.5306 |
| Several times | 14/39 (36) | 1.0 (ref) |  |
| Once | 91/217 (42) | 1.29 (0.64–2.68) |  |
| No | 156/350 (45) | 1.44 (0.73–2.92) |  |
| **The teenager’s level of trust in the care provided to the dying parent in the final week of life** |  |  | **0.0246** |
| Trust (moderate, or very much trust) | 218/485 (45) | 1.66 (1.07–2.62) |  |
| Distrust (no, or a little trust) | 34/103 (33) | 1.0 (ref) |  |
| **The teenager’s perception of whether mistakes had been made in the parent’s care** |  |  | 0.26 |
| No | 173/384 (45) | 1.21 (0.87–1.70) |  |
| Yes | 90/223 (40) | 1.0 (ref) |  |
| **The teenager’s perception of the health care professionals’ efforts to cure the parent** |  |  | **0.0013** |
| Good efforts (moderate, or very much) | 212/451 (47) | 1.84 (1.27–2.71) |  |
| Poor efforts (no, or a little) | 52/160 (32) | 1.0 (ref) |  |
| **The teenager’s perception of the health care professionals’ efforts to prolong the parent’s life** |  |  | **0.0159** |
| Good efforts (moderate, or very much) | 211/459 (46) | 1.59 (1.09–2.34) |  |
| Poor efforts (no, or a little) | 53/152 (35) | 1.0 (ref) |  |
| **The teenager’s perception of the health care professionals’ efforts to prevent suffering** |  |  | **0.0015** |
| Good efforts (moderate, or very much) | 240/524 (46) | 2.18 (1.34–3.67) |  |
| Poor efforts (no, or a little) | 24/86 (28) | 1.0 (ref) |  |
| **Were the family given end-of-life information about the disease, treatment and death by a physician** |  |  | 0.1322 |
| Yes, before, or yes, both before and after the loss | 138/292 (47) | 1.55 (0.91–2.67) |  |
| Yes, after the loss | 13/25 (52) | 1.88 (0.75–4.77) |  |
| Don’t know, don’t remember | 87/223 (39) | 1.12 (0.64–1.94) |  |
| No | 26/71 (37) | 1.0 (ref) |  |
| **The teenager had talked with the dying parent about what was important** |  |  | **<0.0001** |
| Yes | 118/225 (52) | 2.25 (1.57–3.24) |  |
| No, but I didn’t feel a need to | 52/100 (52) | 2.21 (1.39–3.53) |  |
| No, and I wish I had | 92/280 (33) | 1.0 (ref) |  |
| **The teenager had the opportunity to say farewell to the deceased parent** |  |  | 0.0975 |
| Yes, on several occasions | 16/39 (41) | 1.14 (0.48–2.68) |  |
| Yes, at the place of death | 193/457 (42) | 1.20 (0.66–2.21) |  |
| Yes, at another place | 36/62 (58) | 2.26 (1.06–4.90) |  |
| No | 19/50 (38) | 1.0 (ref) |  |
| **The teenager’s perception of the seriousness of the situation 3 days before the loss** |  |  | 0.3248 |
| Already very serious and the end was near | 152/356 (43) | 0.59 (0.25–1.32) |  |
| Serious, incurable disease, but the end was not near | 52/112 (46) | 0.68 (0.28–1.63) |  |
| Serious, but treatment would probably cure the parent | 42/111 (38) | 0.48 (0.20–1.15) |  |
| Not so serious, treatment would cure the parent | 14/25 (56) | 1.0 (ref) |  |
| **The teenager’s location at the time of loss** |  |  | 0.757 |
| With the dying parent | 79/189 (42) | 0.90 (0.62–1.3) |  |
| In a room or corridor next to the dying parent’s room | 37/92 (40) | 0.84 (0.52–1.35) |  |
| On their way to the dying parent | 20/41 (49) | 1.19 (0.62–2.30) |  |
| In school or some other place | 128/288 (44) | 1.0 (ref) |  |
| **Awareness time at which the teenager realized that the parent’s disease was incurable** |  |  | 0.2208 |
| At the time of death | 42/102 (41) | 0.92 (0.58–1.45) |  |
| Hours – days before the death | 61/154 (40) | 0.86 (0.58–1.28) |  |
| Weeks – months before the death | 125/289 (43) | 1.0 (ref) |  |
| 6 months or longer before the death | 36/66 (55) | 1.57 (0.92–2.71) |  |
| **Awareness time at which the teenager realized that the parent would die from the disease** |  |  | 0.0917 |
| At the time of death | 51/123 (41) | 0.84 (0.54–1.29) |  |
| Hours – days before the death | 73/193 (38) | 0.72 (0.49–1.05) |  |
| Weeks – months before the death | 111/242 (46) | 1.0 (ref) |  |
| 6 months or longer before the death | 28/50 (56) | 1.50 (0.82–2.80) |  |
| **Awareness time at which the teenager realized that death was imminent (hours or days)** |  |  | 0.3953 |
| At the time of death, or never | 77/191 (40) | 1.0 (ref) |  |
| Hours to 2 days before the death | 126/293 (43) | 1.13 (0.77–1.62) |  |
| 3 days or longer before the death | 61/127 (48) | 1.37 (0.87–2.15) |  |

^1^Associations between having had an okay way to grieve and various background, family or health care-related variables,

assessed with bivariable logistic regression.

CI = confidence interval; OR = odds ratio.
